# Interaction of Genotype, Environment, and Management on Organ-Specific Critical Nitrogen Dilution Curve in Wheat

**DOI:** 10.34133/plantphenomics.0078

**Published:** 2023-08-02

**Authors:** Bo Yao, Xiaolong Wang, Yancheng Wang, Tianyang Ye, Enli Wang, Qiang Cao, Xia Yao, Yan Zhu, Weixing Cao, Xiaojun Liu, Liang Tang

**Affiliations:** ^1^ National Engineering and Technology Center for Information Agriculture, Engineering Research Center for Smart Agriculture, Ministry of Education, Key Laboratory for Crop System Analysis and Decision Making, Ministry of Agriculture and Rural Affairs, Jiangsu Key Laboratory for Information Agriculture, Jiangsu Collaborative Innovation Center for Modern Crop Production, Nanjing Agricultural University, Nanjing 210095, Jiangsu, PR China.; ^2^ CSIRO Agriculture and Food, Cluniess Ross Street, Black Mountain, ACT, Australia.

## Abstract

The organ-specific critical nitrogen (N_c_) dilution curves are widely thought to represent a new approach for crop nitrogen (N) nutrition diagnosis, N management, and crop modeling. The N_c_ dilution curve can be described by a power function (N_c_ = A_1_·W^−A2^), while parameters A_1_ and A_2_ control the starting point and slope. This study aimed to investigate the uncertainty and drivers of organ-specific curves under different conditions. By using hierarchical Bayesian theory, parameters A_1_ and A_2_ of the organ-specific N_c_ dilution curves for wheat were derived and evaluated under 14 different genotype × environment × management (G × E × M) N fertilizer experiments. Our results show that parameters A_1_ and A_2_ are highly correlated. Although the variation of parameter A_1_ was less than that of A_2_, the values of both parameters can change significantly in response to G × E × M. Nitrogen nutrition index (NNI) calculated using organ-specific N_c_ is in general consistent with NNI estimated with overall shoot N_c_, indicating that a simple organ-specific N_c_ dilution curve may be used for wheat N diagnosis to assist N management. However, the significant differences in organ-specific N_c_ dilution curves across G × E × M conditions imply potential errors in N_c_ and crop N demand estimated using a general N_c_ dilution curve in crop models, highlighting a clear need for improvement in N_c_ calculations in such models. Our results provide new insights into how to improve modeling of crop nitrogen–biomass relations and N management practices under G × E × M.

## Introduction

Wheat is widely acknowledged as a key cereal crop for global food security. However, due to increasing wheat demand and decreasing arable land, further increase in yield is necessary to ensure food security. Nitrogen (N) fertilizers are widely used to match the N demand for high-yielding wheat crops. However, excessive N fertilizer input in agricultural systems can negatively affect wheat growth [1] and pollute the environment, leading to low nitrogen use efficiency and waste of resources [2]. Therefore, crop N diagnosis, crop N requirement estimation, and improved N fertilizer management are crucial to improving wheat yield and N use efficiency [3].

Estimating crop N demand requires information about crop growth and the N nutritional status of crop, which is generally estimated based on the critical N concentration (N_c_). N_c_ is the minimum N concentration required to meet the maximum growth of crops [4]. Lemaire and Salette [5] proposed the concept of the N_c_ dilution curve. The N nutrition index derived from the N_c_ dilution curve has been considered as a reliable tool for quantifying plant N status, optimizing fertilization strategies, and estimating crop yield [6]. Over the years, more than 30 crops have been evaluated, including rice [7–10], wheat [11,12], maize [13,14], potato [15], and oilseed rape [16].

Most N_c_ dilution curves developed up to now have been based on shoot biomass. However, leaf is the major photosynthetic organ that is highly responsive to N application [17,18], while stem biomass dominates shoot biomass at late growth stage. Plants maintain higher leaf N concentration throughout the vegetative period for optimal photosynthesis, with reduced N concentrations in shaded leaves in a closed canopy. Current evidence suggests that distribution patterns of assimilates in different organs are altered by N stress. Consequently, the shape of the N_c_ dilution curve vary depending on the plant organs [19]. Zhao et al. [20] and Yao et al. [21,22] developed N_c_ dilution curves based on leaf biomass of rice, winter wheat, and maize. The curves for wheat based on leaf biomass (A_1_ = 3.06; A_2_ = 0.15) were lower than those based on shoot biomass [11,12]. Interestingly, a study found that compared with irrigation conditions, rainfed wheat had lower leaf critical N in southeastern China [23]. N_c_ dilution curves based on stem biomass generally yield lower initial values and steeper declines [23,24]. The variability of curve parameters indicates that it is affected by the interaction of genotype (G), environment (E), and management (M), which embody sources of the uncertainty in model parameter estimation [25,26]. Ata-Ul-Karim et al. [24] compared the nitrogen nutrition index (NNI) derived from N_c_ dilution curves based on the shoot, organ biomass, and leaf area index (LAI) in rice, and found that NNI derived from shoot and leaf biomass was the most relevant. Sieling and Kage [27] indicated that the NNI derived from different organ and shoot biomass was inconsistent across oilseed rape, winter wheat, and maize under different N nutrition levels.

In recent years, the uncertainty of agricultural models and the uncertainty of fitted N_c_ dilution curves have received extensive attention. For analysis of N_c_ dilution curves, the often used classical sequential methods [11] have limitations and cannot estimate the error of the fitted curves. An alternative method to analyze the uncertainty and difference of N_c_ dilution curve parameters for major field crops (rice, wheat, and maize) was proposed based on Bayesian theory [28], which can directly fit the curve from crop biomass and N concentration observations in one step. Using this method, the uncertainties of N_c_ dilution curves of maize [29], wheat [25], tomato [30], and tall fescue [31] under different G × E × M conditions have been evaluated.

For crop models that rely on N_c_ curves to determine crop N demand, the parameters may need to be updated because most existing crop models were established more than 20 years ago. They were developed using a small dataset and the N_c_ curves were mostly developed based on shoot biomass. To our knowledge, no study has hitherto reported the uncertainty of the N_c_ dilution curve based on wheat organs, nor is it clear whether the NNI derived from different N_c_ dilution curves is consistent.

Here, we use data from 14 wheat N fertilizer experiments under different G × E × M conditions to develop and compare organ-specific N_c_ dilution curves. We further analyze the uncertainty of the organ-based (leaf, stem biomass, and LAI) N_c_ dilution curves, variations in their parameters, and the source of uncertainty under different experimental (G × E × M) conditions. Finally, we compare the differences in different organ-based NNI.

## Materials and Methods

### Experiment design

Fourteen wheat N experiments were carried out in central and eastern China at 5 sites: Yizheng, Rugao, Xinxiang, Xuzhou, and Sihong (Table S1). Five widely planted wheat genotypes (Jimai 22, Xumai 30, Ningmai 13, Aikang 58, and Yangmai 16) were used to evaluate the N_c_ dilution curves. All the experiments involved different N application rates from 0 kg N/ha to a high N rate of >300 kg N/ha. Except for experiment 5 (with 3 N rates), at least 5 N fertilizer application rates were used in all other experiments. N fertilizer was split into 2 applications, one before sowing and the other at the jointing stage. The field management followed the local recommendations, with sufficient phosphorus and potassium fertilizer application, irrigation if necessary to avoid water stress, and proper control of diseases, pests, and weeds. Table S1 shows the details of the experimental design, Table S2 shows the information of the experiment site, and Fig. S1 shows the meteorological conditions.

### Plant sampling and N concentration

In each experiment, wheat plant was sampled at least 5 times before flowering to evaluate the N_c_ dilution curve (Table S3). The leaf area was immediately measured for fresh leaves to calculate the LAI (device: LI-3000, Li-COR, Lincoln, USA). Plant samples were separated into organs (leaves and stems), and tissue metabolism was deactivated at 105 °C in an oven for 30 min. Then, they were dried to constant weight at 80 °C to calculate biomass. The dried samples were mechanically crushed and passed through a 2-mm sieve, and then the organ N concentration was determined by the Kjeldahl method [32].

### Data analysis

#### Parameter estimation

**Fig. 1.**
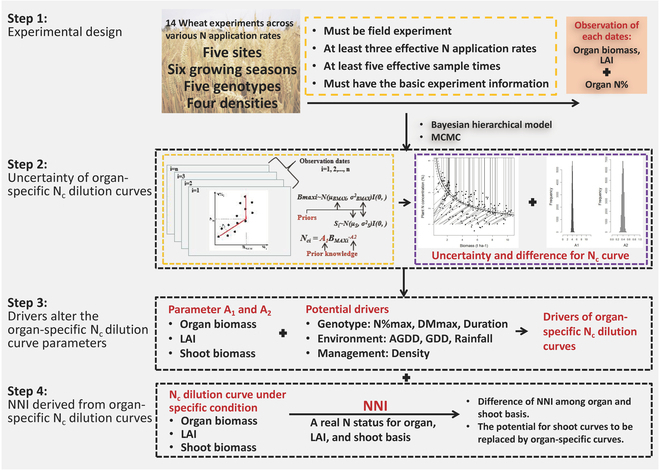
Experiments and technical flowchart of this study.

Organ-specific N_c_ dilution curves were evaluated by a hierarchical Bayesian model [28], which follows the response of biomass or LAI to N concentration (organ or shoot N concentration) as a linear-plus-plateau function [11,13]. According to the probability distribution estimated by the Bayesian method, the uncertainty of parameters of the linear-plus-plateau function at different observation dates (at the biomass or LAI level) can be analyzed. For the classical method, a N_c_ dilution curve can be estimated only after variance analysis and curve fitting, and the uncertainty of the whole process cannot be determined. The advantage of the Bayesian method is that the parameters and uncertainty of the N_c_ dilution curve are directly from the probability distribution. Therefore, the difference of curve parameters under different G × E × M conditions can be compared (Fig. 1).

In this study, the range of prior information is adjusted without strong restrictions so that a stable posterior distribution can be obtained. The prior ranges of organ-specific curve parameters A_1_ and A_2_ were 0 to 12 and 0 to 5, respectively. The other settings were consistent with Makowski et al. [28]. The fitted curves that were not convergent in Markov chain Monte Carlo (MCMC) were deleted.

$$N_{c}=A_{1}\cdot W^{-A_{2}}$$ (1)

where N_c_ is the critical N concentration of organs and shoot, and W is the biomass of organs and shoot and LAI. A_1_ and A_2_ are the N_c_ dilution curve parameters. The R package “rjags” was used to fit the posterior distribution of curve parameters with the MCMC algorithm [33]. The MCMC algorithm first iterated about 100,000 times to achieve convergence, and the algorithm continued to be run 30,000 times to analyze multiple quantities of interest (median and 95% credible interval). The N_c_ dilution curves based on stem, leaf, and shoot biomass and LAI were derived from stem biomass and stem N concentration, leaf biomass and leaf N concentration, shoot biomass and shoot N concentration, and LAI and shoot N concentration, respectively.

#### Significance analysis

The generalized linear model was used for analysis of variance with SPSS16.0 software. Differences between the posterior curve parameters (A_1_ and A_2_) were compared by Tukey significant difference test at the 0.05 probability level.

#### Correlation

The R package “Hmisc” was used to calculate the correlation matrix to determine the source of parameter differences [34]. The relationship between N_c_ dilution curve parameters (A_1_ and A_2_) under different G × E × M conditions and corresponding G, E and M information was comprehensively analyzed. The G × E × M information included the maximum shoot biomass (DMmax), maximum shoot N concentration (Nmax) during the vegetative growth period, vegetative growth period duration (VPD), accumulated growth degree days (AGDD), daily average GDD, and rainfall during the vegetative growth period and planting density (Fig. 1).

$$\text{AGDD}=\sum_{1}^{N}\left(\frac{T_{\max}+T_{\min}}{2}-T_{\mathrm{base}}\right)$$ (2)

where N is the duration of the vegetative growth period in days, T_base_ is the lowest temperature for wheat to start physiological activities, and T_min_ and T_max_ are the lowest and highest temperature of the day, respectively; the T_base_ is 0 °C in this study [35,36].

#### Nitrogen nutrition index

The NNI for each observation point was calculated as follows [11]:

$$\mathrm{NNI}=\frac{N_{t}}{N_{c}}$$ (3)

where NNI is the ratio between measured N concentration (N_t_) and critical N concentration (N_c_) of different organ or shoot biomass for each sampling date.

The RMSE (root-mean-square error) and n-RMSE (normalized root-mean-square error) were calculated as follows:

$$\mathrm{RMSE}=\sqrt{\frac{\sum_{i-1}^{n}{\left(P_{i}-O_{i}\right)}^{2}}{n}}$$ (4)

$$n-\mathrm{RMSE}=\frac{\mathrm{RMSE}}{S}\times 100\%$$ (5)

where n is the data size, O_i_ and P_i_ are the NNI derived by the hybrid N_c_ dilution curve and the specific N_c_ dilution curve, and S is the average value for the data.

#### Coefficient of variation

$$\mathrm{CV}=\frac{\mathrm{SD}}{\mathrm{MN}}\times 100\%$$ (6)

where CV is the coefficient of variation, SD is the standard deviation, and MN is the mean value.

## Results

### Variations in organ-specific N_c_ dilution curve parameters

**Fig. 2.**
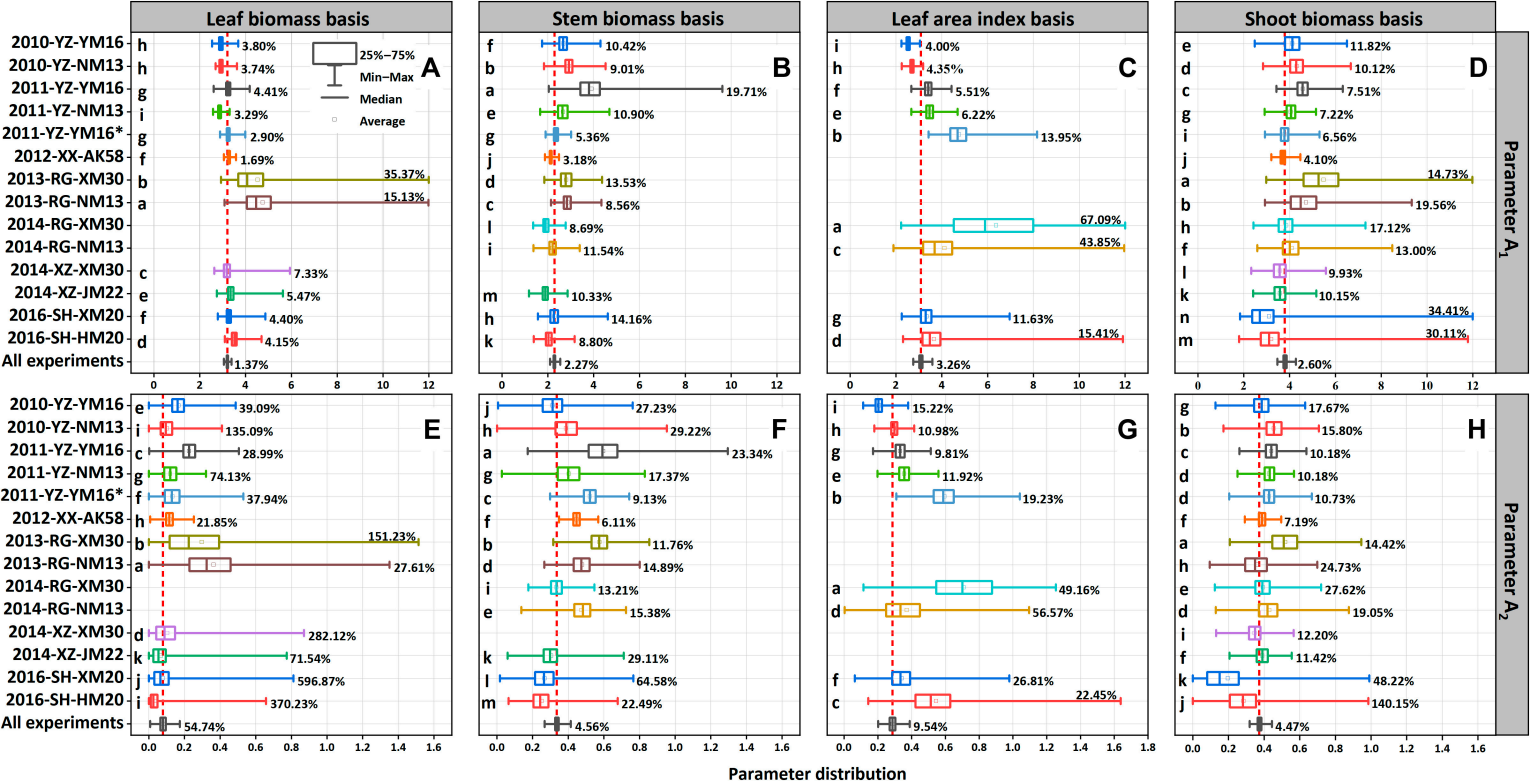
The posterior distribution of curve parameters (A_1_ and A_2_) for wheat organs across different G × E × M conditions. The significant difference (*P* < 0.05) is represented by different letters. The CV of the fitted curve parameters (A_1_ and A_2_) is represented by percentage.(A, E) Leaf biomass basis; (B, F) stem biomass basis; (C, G) leaf area index basis; (D, H) shoot biomass basis.

The posterior parameter distribution of the N_c_ dilution curves for the organs, shoot biomass, and LAI are shown in Figs. S2 to S5, and Tables S4 to S7 describe the quantiles of the posterior distribution of parameters, with non-convergent curve parameters deleted. Only a few curves exhibited the same curve parameters, and there were significant differences in fitted curves derived using the 4 different types of data, across genotypes and planting environment (Fig. 2). Notably, cultivation under the same planting sites and years and management yielded differences across genotypes. Significant differences were observed in parameters for a given genotype under different environment × management conditions, indicating that environment × management has significant effects on parameters A_1_ and A_2_. For parameter A_1_, 6 curves based on leaf biomass have the same A_1_ value (*P* > 0.05, Fig. 2A). Curves based on stem, shoot biomass, and LAI yielded significantly different values (*P* < 0.05). For parameter A_2_, two curves based on leaf and shoot biomass have the same value (*P* > 0.05, Fig. 5E and H). For stem biomass and LAI, no curve yielded the same value (*P* < 0.05), which indicates that the curve based on leaf biomass may be subject to less variation under different G × E × M conditions.

The values of the curve parameters A_1_ and A_2_ mean the starting position and the dilution rate of the curve, respectively. The CVs between the effects of the G × E × M interaction of parameter A_2_ were higher than parameter A_1_. In particular, the CV of parameter A_1_ based on leaf biomass was much smaller than that of parameter A_2_. For parameter A_1_, it has the least variation with leaf biomass, but the greatest variation with shoot biomass. For parameter A_2_, it has the least variation with LAI, but the greatest variation with leaf biomass. More experimental observations also made the variation in the posterior distribution of the parameters smaller.

### The drivers in organ-specific N_c_ dilution curve parameters

**Fig. 3.**
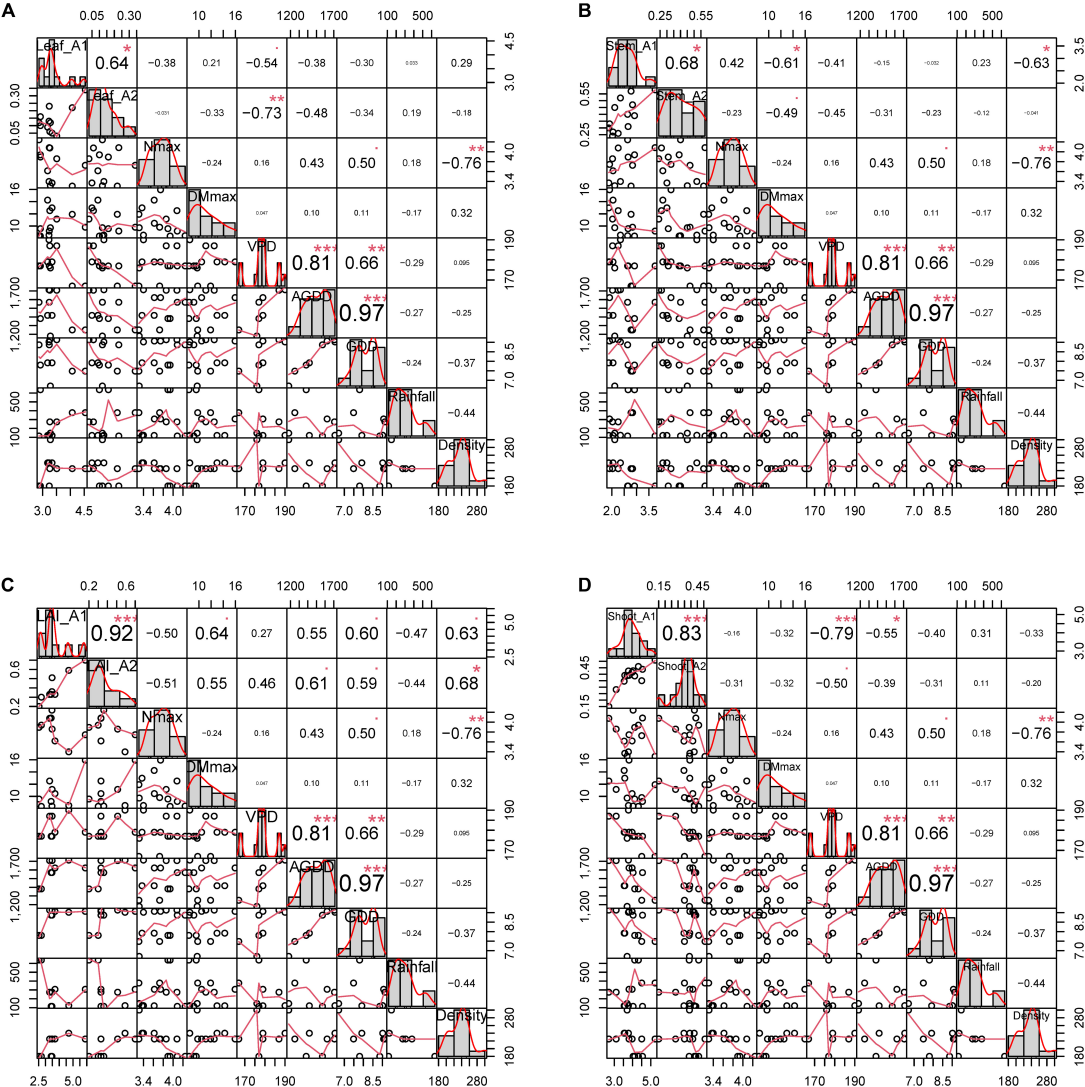
Correlation and significance matrix between the fitted parameter (A_1_ and A_2_) for different wheat organs across genotypes, environments, and managements. (A) Leaf biomass basis; (B) stem biomass basis; (C) leaf area index basis; (D) shoot biomass basis. ·, *P* < 0.1; *, P < 0.05; **, P < 0.01; ***, P < 0.001. Correlation coefficient values with bigger font sizes show more significant correlations.

The curve parameter A_1_ based on leaf, stem, shoot biomass, and LAI was significantly correlated with parameter A_2_ (*P* < 0.05, Fig. 3). The initial maximum shoot N concentration (Nmax), which is the characteristic of the wheat genotype, has no significant correlation with the curve parameters for different organs (*P* > 0.1). Similarly, the maximum shoot biomass (DMmax) during the vegetative period was only negatively correlated with the curve parameter A_1_ and A_2_ for the stem biomass (*P* < 0.1, Fig. 3B), which indicated that the fitted curve parameters might be less affected by genotype differences. Interestingly, VPD was significantly negatively correlated with curve parameters (A_1_ and A_2_) for shoot and leaf biomass basis (Fig. 3A and D), but it was not significantly correlated with curve parameters (A_1_ and A_2_) for stem biomass and with LAI basis (*P* > 0.1, Fig. 3B and C). For the environmental characteristics involved in the analysis, there was no significant correlation between rainfall and the above curve parameters (*P* > 0.1). AGDD was only significantly correlated with the parameter A_1_ for shoot biomass basis and A_2_ for LAI basis (*P* < 0.05, Fig. 3C and D). The average daily GDD was significantly correlated with parameters A_1_ and A_2_ for LAI (*P* < 0.1, Fig. 3C). Notably, AGDD significantly correlated with VPD (*P* < 0.001). Only sowing density was included in the analysis as management information, and curve parameter A_1_ for stem biomass was significantly negatively correlated with sowing density (*P* < 0.05, Fig. 3B). However, it was significantly positively correlated with curve parameters A_1_ and A_2_ for LAI (*P* < 0.05, Fig. 3C). Overall, genotype affects curves based on LAI and stem and shoot biomass, but not leaf biomass. Environment affects all curves except for leaf biomass. Density affects all curves, except for leaf biomass.

### Variation in organ-specific N_c_ dilution curves and uncertainty in parameters

**Fig. 4.**
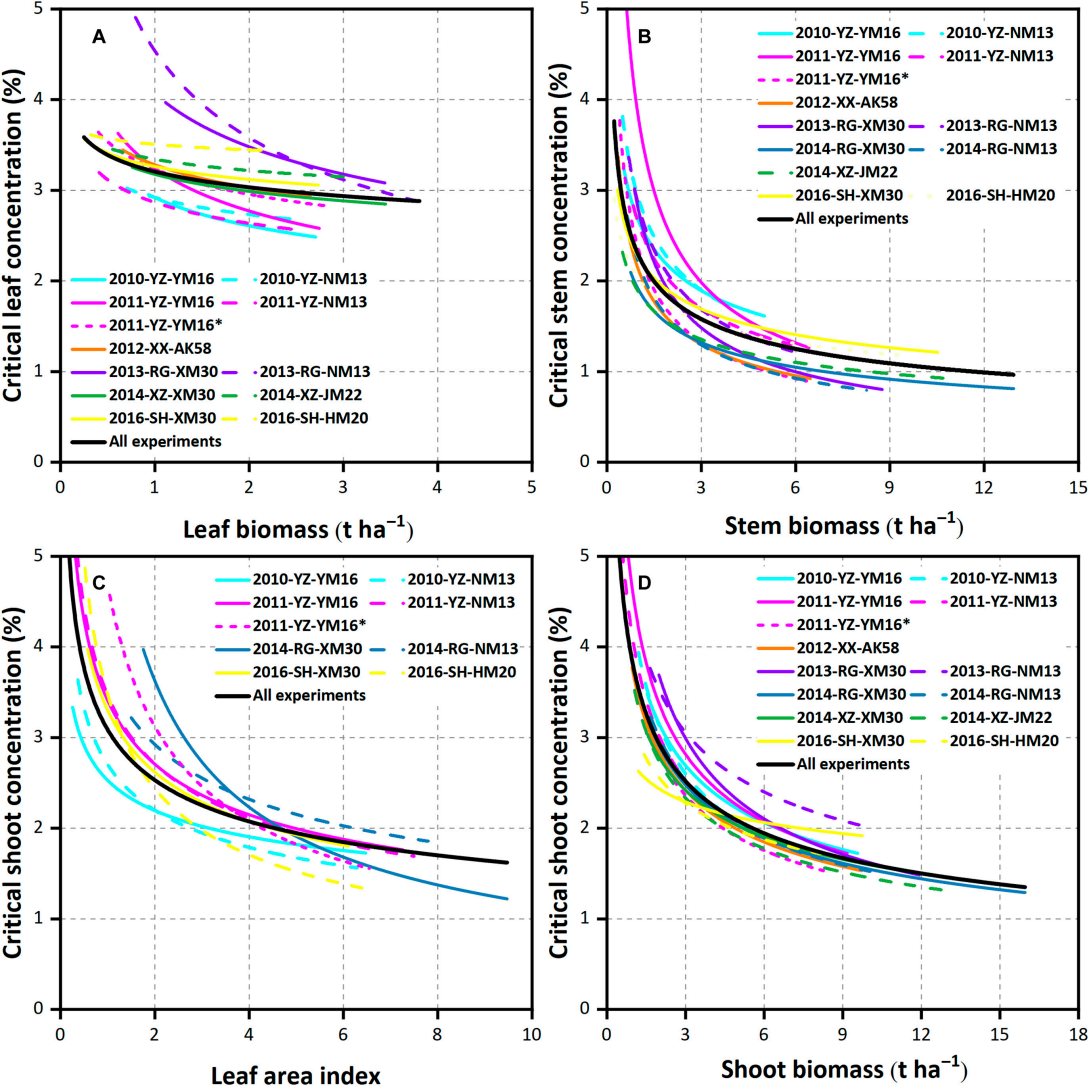
The critical N dilution curve for different wheat organs under different G × E × M conditions. (A) Leaf biomass basis; (B) stem biomass basis; (C) leaf area index basis; (D) shoot biomass basis.

N_c_ dilution curves based on leaf, stem, shoot biomass, and LAI under 14 G × E × M conditions are shown in Fig. 4. N concentration decreased with the increase of biomass and LAI. The leaf maintained a high N_c_ concentration during the observation period and exhibited mild decreases with increased leaf biomass. The leaf N_c_ concentration level under different experimental conditions exhibited significant differences (Fig. 4A). However, the N_c_ concentration of the stem was lower than the leaf and decreased faster with increased stem biomass. The differences in the N_c_ concentration of stem under different experimental conditions were less than the leaf (Fig. 4B). The N_c_ curve of the shoot biomass was higher than the stem biomass. There was a small difference in the N_c_ curves of the shoot biomass under different experimental conditions (Fig. 4D). In addition, the N_c_ curve for the LAI basis was higher than the shoot biomass (Fig. 4C and D). There also was a substantial difference in the N_c_ curves for LAI under different experimental conditions.

**Fig. 5.**
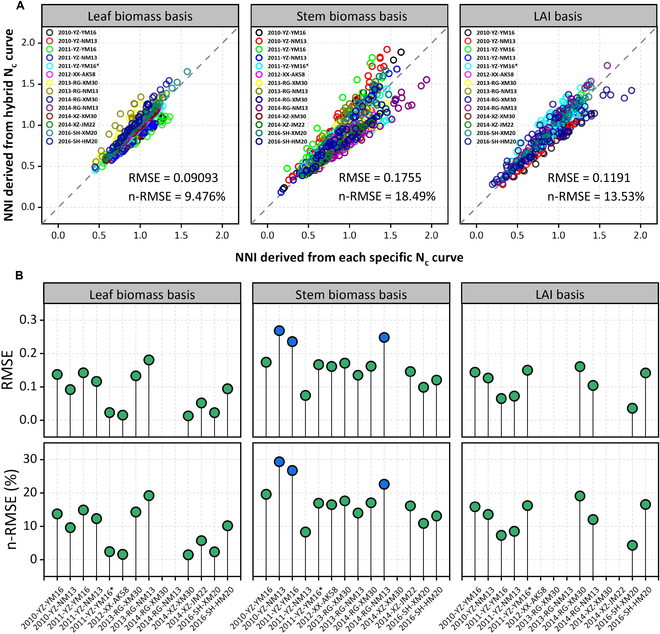
Comparison of NNI derived from the hybrid organ-specific N_c_ curves and each organ-specific curve under different conditions. (A) Verification of all experimental data; (B) verification of each experimental data.

**Fig. 6.**
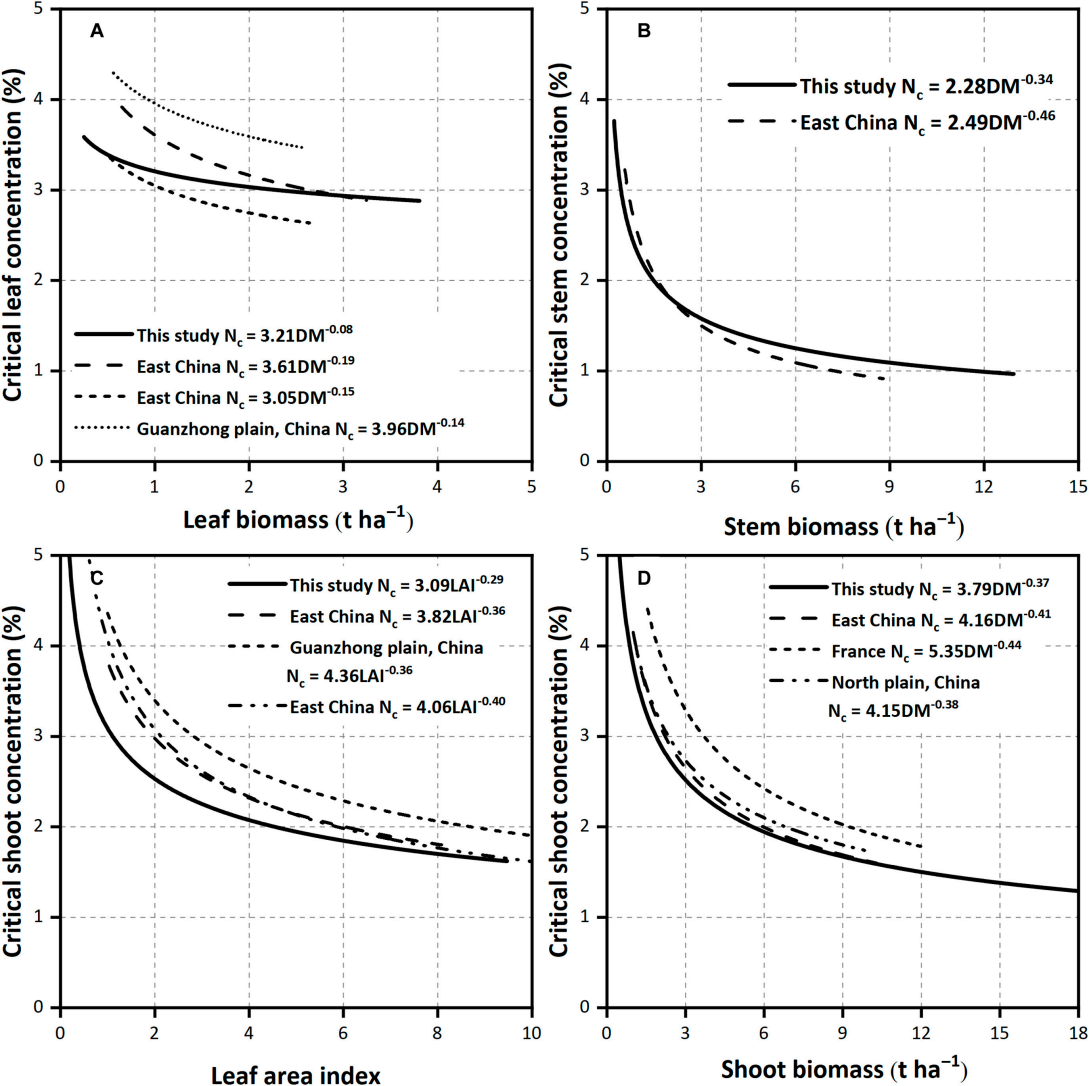
The published curves and organ-specific curves estimated globally (posterior medians). (A) Leaf biomass basis; (B) stem biomass basis; (C) leaf area index basis; (D) shoot biomass basis.

The hybrid organ-specific N_c_ dilution curves were fitted across all experimental data points (Fig. 4, black lines). The difference between the NNI derived from organ-specific curves under specific conditions and the NNI derived from corresponding hybrid curves was relatively small (Fig. 5). For NNI derived from leaf and stem biomass, and LAI, the n-RMSE values were 9.48%, 18.49%, and 13.53%, respectively (Fig. 5). This indicated that the errors were statistically acceptable if the organ N status was estimated using the universal curve. The N_c_ dilution curves based on organs, shoot biomass, and LAI were compared with the published N_c_ dilution curve (Fig. 6); the curve parameter A_1_ for leaf biomass basis was in between the comparison curves [22,23,37,38]; the curve parameter A_1_ for stem biomass [23,38], LAI, and shoot biomass basis [11,12] was lower than the comparison curve [38]. The curve parameter A_2_ for organs, shoot biomass, and LAI basis was lower than all comparison curves.

**Fig. 7.**
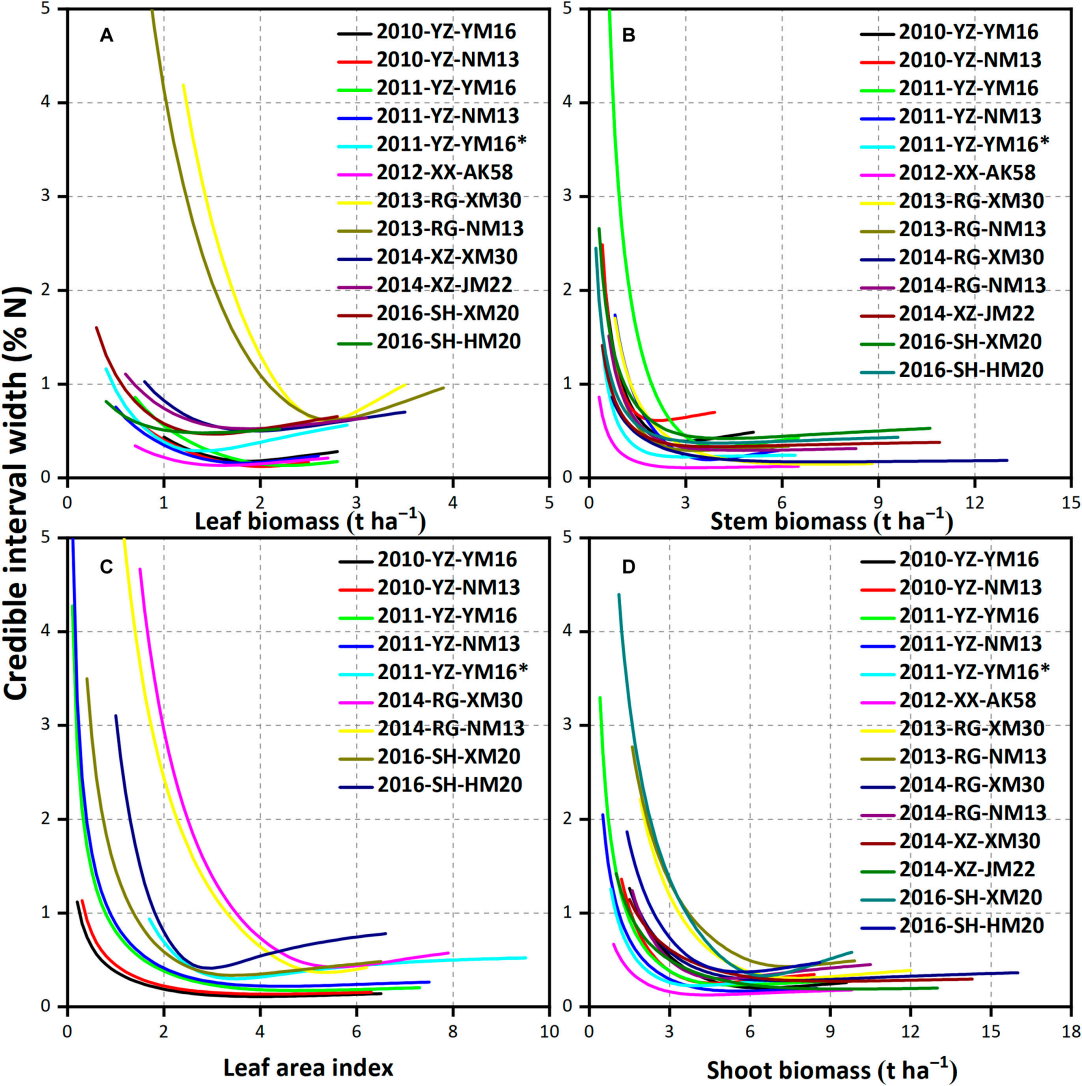
The credible interval width of critical N dilution curve for different wheat organ bases. (A) Leaf biomass basis; (B) stem biomass basis; (C) leaf area index basis; (D) shoot biomass basis.

The uncertainty of the N_c_ curve (the 95% credible interval width) based on organs, shoot biomass, and LAI is shown in Fig. 7. The uncertainty decreased rapidly when the biomass or LAI was low and then remained stable or rose slightly. However, some curves showed a narrow credible interval. Furthermore, it is well-established that more observation points can reduce the uncertainty of the N_c_ curve. In this study, the experiment 2012-XX-AK58 (*n* = 91) included 13 N fertilizer treatments, including 5 N application rates, 3 topdressing ratios, and 7 sampling dates. Under higher biomass and LAI (leaf biomass > 2 t ha^−1^, stem biomass > 3 t ha^−1^, LAI > 2.5, and shoot biomass > 5 t ha^−1^), the uncertainty was least in shoot biomass, followed by stem biomass, LAI, and leaf biomass.

### NNI derived from organ-specific N_c_ dilution curves

The NNI for leaf, stem, shoot biomass, and LAI basis was subsequently calculated (Fig. S6). The NNI for each organ basis at different stages was increased with an increased N fertilizer rate. The NNI for leaf biomass basis maintained a higher NNI value (mean = 0.84) during the vegetative growth period across several N treatments, and the CV was smaller (CV = 22.83%). Compared with leaf, the CV of NNI of stem biomass basis was larger (40.45%) and maintained a lower value (mean = 0.74). Notably, the N_c_ dilution curves for LAI and shoot biomass were derived from shoot N concentration. However, the NNI value of the LAI was higher (mean = 0.72) than the shoot biomass (mean = 0.69), and the CV was larger (CV = 43.28%).

**Fig. 8.**
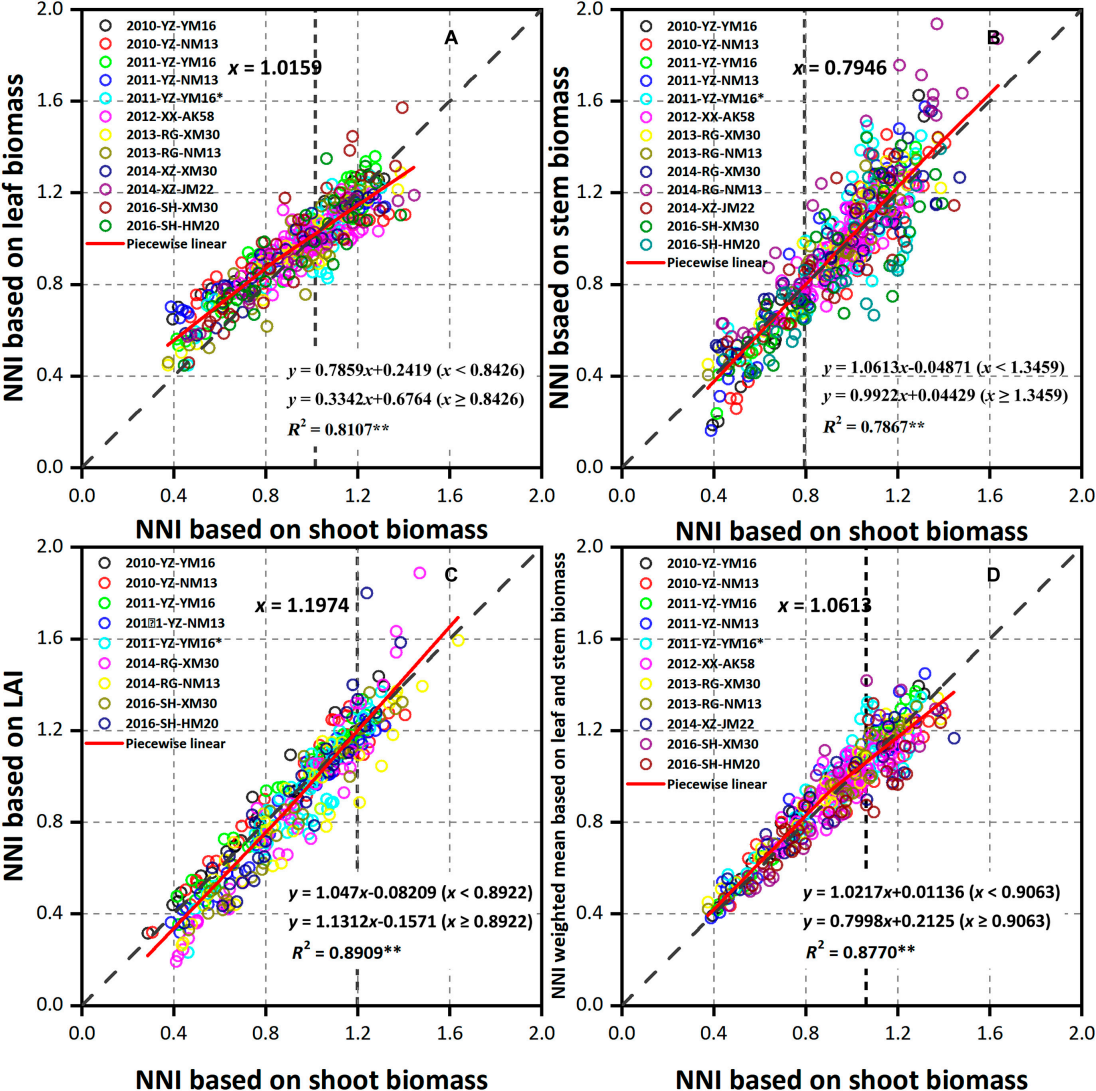
Comparison of the N nutrition index (NNI) derived from different organ and shoot biomass. (A to C) The relationship between NNI based on leaf biomass, stem biomass, and leaf area index and NNI based on shoot biomass, respectively. (D) The relationship between NNI weighted mean based on leaf biomass, stem biomass, and NNI based on shoot biomass.

The relationship between the NNI for leaf, stem biomass, and LAI and the NNI for shoot biomass in each experiment is shown in Fig. 8. When the shoot-based NNI exceeded 0.84, the increase in leaf NNI decreased; when the shoot NNI exceeded 1.02, the leaf NNI was lower than the 1:1 line. In contrast, when the shoot NNI exceeded 0.79, the stem NNI was relatively higher. Moreover, when the shoot NNI exceeded 1.35, the stem NNI showed a more significant increase rate. LAI NNI was similar to stem NNI, but the LAI NNI was higher than the 1:1 line when the shoot NNI exceeded 1.20. When the shoot NNI exceeded 0.89, the increased rate of LAI NNI increased. In addition, the correlation between stem NNI and shoot NNI was low (*R*^2^ = 0.7867). These findings indicated that the N status of different organs was different from the shoot.

**Fig. 9.**
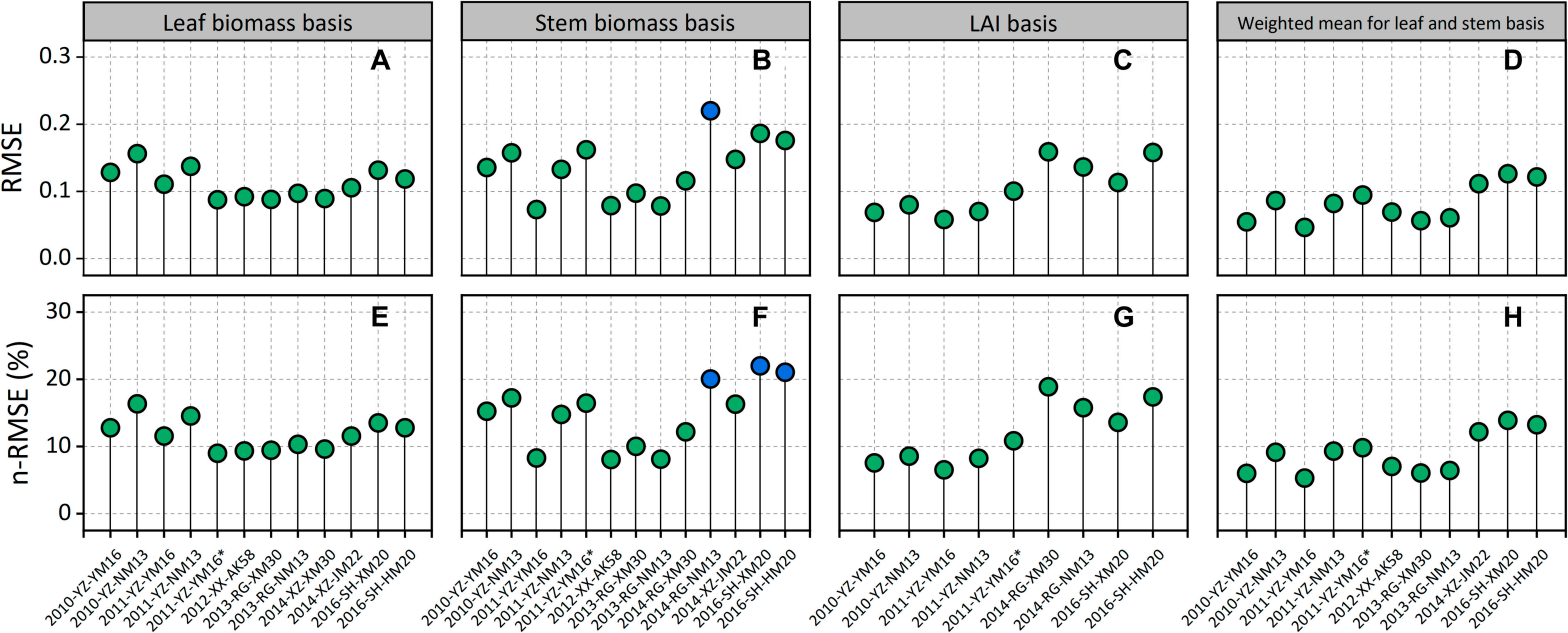
Comparison of the N nutrition index for different organ bases and shoot biomass basis for each wheat experimental condition. (A to C) The difference between NNI based on leaf biomass, stem biomass, and leaf area index and NNI based on shoot biomass, respectively. (D) The difference between NNI weighted mean based on leaf biomass, stem biomass, and NNI based on shoot biomass.

Overall, leaf biomass NNI yielded the smallest difference with shoot NNI (RMSE = 0.1094; n-RMSE = 11.39%), followed by LAI NNI (RMSE = 0.1096; n-RMSE = 12.36%), while stem NNI yielded the largest difference (RMSE = 0.1384; n-RMSE = 14.63%). However, there was little difference between different organs and shoot N status (n-RMSE < 15%). For each specific experimental condition (Fig. 9), the difference between leaf biomass NNI and LAI NNI and plant NNI was in RMSE < 0.2 and n-RMSE < 20%. In some experiments, the difference between stem NNI and shoot NNI was more than 20% (n-RMSE). Notably, the difference between shoot NNI and weighted mean NNI for leaf and stem basis was very small.

## Discussion

### Drivers altering the organ-specific N_c_ dilution curve parameters

Most N_c_ dilution curves fitted for organ biomass and LAI bases are different, reflecting the effect of G × E × M interaction. This implies that changes of wheat genotypes, planting environment, and management can affect the curve parameters, leading to potential errors in crop N nutrition status diagnosis and N demand estimations [28,39,40]. The uncertainty of the fitted parameter A_1_ was significantly less than that of parameter A_2_, indicating that the curve parameter A_1_ for organ basis was less affected by G × E × M. The organ-specific N_c_ dilution curves showed that the N concentration in the leaf for all experiments was higher than in the stem (Fig. 4), mainly due to the different demands of plant organs on metabolic and structural N [41]. The wheat leaf N concentration decreased slightly (almost linearly) during the whole vegetative growth stage due to the effective utilization of physiological N in the leaves to ensure optimal photosynthetic activity [18,24]. The accurate estimation of the N_c_ dilution curve can reflect the real difference under different conditions [6] and allow to find the source of the difference [25,28,29].

For the N_c_ dilution curves for leaf biomass, parameters A_1_ and A_2_ were negatively correlated with the VPD. The proportion of the lower leaves increased and were shaded by the upper leaves. Accordingly, the difference of light environment and leaf age may lead to this dilution phenomenon [19,42–45]. Notably, the datasets ignored the changes of N concentration under initial low biomass (shoot biomass <1 t ha^−1^), which is a more standardized method according to Justes et al. [11]. Therefore, all curve parameters developed based on organ biomass and LAI had no significant correlation with plant N concentration during the early growth stage. For the N_c_ dilution curves for stem biomass basis, parameters A_1_ and A_2_ were negatively correlated with the maximum biomass in the vegetative growth period. The N concentration dilution was much stronger in stem and shoot during the early dilution process [23]. It is widely acknowledged that stem biomass accounts for a major component of shoot biomass. The negative correlation between parameter A_1_ and planting density indicated that high density affected N uptake by the stem during the early growth stage. In addition, planting density was positively correlated with curve parameters A_1_ and A_2_ for LAI, and wheat canopy coverage was changed by planting density, wheat plants could quickly enter the dilution process under high density, and plant biomass increased faster [14,46]. Parameter A_2_ reflects a specific mode of plant N concentration dilution with the increase of biomass. Our results indicated that the change degree of curve parameter A_1_ was lower than parameter A_2_ [7,11,14,19]. Because all experiments were irrigated when necessary, the significant effect of rainfall on parameters A_1_ and A_2_ of the organ-specific curve was not determined. The effect of the G × E × M interaction was slightly greater than parameter A_1_, and its planting environment easily determined the response of biomass or LAI to N uptake during wheat growth.

### Universal organ-specific N_c_ dilution curves

The classic method of developing N_c_ dilution curves is obtained through analysis of variance and a series of curve fitting [11,47]. Therefore, the error of the whole process cannot be considered. The datasets under different G × E × M conditions are particularly important for the uncertainty analysis. This study used Bayesian theory and the MCMC method to test the wheat N fertilizer experimental dataset under 14 different G × E × M conditions, and an N_c_ dilution curve based on the wheat leaf, stem, and shoot biomass, and LAI was proposed [28]. The published curves were not consistent with the curve constructed based on the Bayesian theoretical framework in this study [11,12,21,23,37,38,48], which showed the difference in wheat N dilution under different wheat genotypes, planting environments, and managements, and also reflected that the traditional methods might overestimate the N_c_ concentration [31], given that part of the experimental data used in this study came from a previous study [38]. Indeed, the difference in wheat genotypes leads to differences in N uptake capacity, and this difference may also be related to wheat's initial N uptake capacity during the seedling stage and the soil N supply [41].

Similar to the N_c_ dilution curve for shoot biomass basis [25,28,29], the uncertainty of the N_c_ dilution curve for leaf and stem biomass and LAI basis is related to the biomass or LAI level. The response curve of biomass or LAI to N% was steep at low biomass levels. The uncertainty of N_c_ became less as the response curve asymptotically approached horizontal with higher biomass or LAI. Therefore, the early less accurate N_c_ value was unreliable for decision-making. Besides, the uncertainty level of the fitting curve was related to the experimental dataset. Indeed, the curve parameters can be accurately estimated by an effective dataset, and the real difference of the curve parameters can be obtained [25]. Accordingly, effective experimental design, such as more and reasonable N application treatments, will reduce the uncertainty of each observation date (by reducing data point scattering). Meanwhile, increasing the number of observations (by increasing sampling frequencies) will reduce the uncertainty in the time series [49].

Too many N_c_ dilution curves under specific conditions are undoubtedly not conducive to practical application. Therefore, it is necessary to simplify the model while ensuring the performance of N status estimation. This study proposed the potential for establishing universal organ-specific N_c_ dilution curves. The results indicated that the universal curves have good nitrogen diagnostic ability under different conditions, which is consistent with Yao et al. [25] and Fu et al. [50]. The applicability of the universal curve is expected to be tested in the future.

### NNI derived from organ-specific N_c_ dilution curves and application

The correlations between organs and plant N status make it possible to use organ N status to estimate shoot N status. Previous studies have shown that the NNI based on rice leaf biomass was closer to the shoot biomass basis [24], and the NNI based on wheat leaf biomass was better for N diagnosis [38]. In particular, the N status for leaf biomass and LAI basis was synchronous and stable with the shoot biomass (Fig. 9). The weighted average NNI for leaf and stem biomass basis was closer to the NNI for shoot biomass basis, indicating that leaf and stem were not balanced in the N distribution of the whole plant, which provided a reference for simulating the N distribution among different organs of wheat crops [37]. It was worth noting that leaves started to consume N extravagantly when the shoot N status was not very high (NNI > 0.84; Fig. 8), while stems started to serve as N storage organs when the shoot N status was very high (NNI > 1.35). Indeed, wheat plants first use enhanced N supply to increase their leaf N concentration to optimize photosynthesis, and the stem is then used as a temporary storage organ [27]. For the same observation sample, there were differences in N status among leaf, stem, and shoots. Therefore, the plant and organ N status should be reevaluated in practical application. The difference in organ N status can improve our understanding of organ function response to N nutrition.

At present, many crop growth models use organ N_c_ dilution curves to estimate organ N distribution. The N_c_ curve for APSIM (Agricultural Production Systems sIMulator) [37], STICS (Simulateur Multidisciplinaire pour les Cultures Standards) [51], and RiceGrow [52] models has been developed using a small dataset or special planting conditions, which have yielded great uncertainty. Especially for crop growth modeling, this difference in N dilution effects at the organ level seems to be a useful and logical method for describing their different roles as storage pools and assimilation organs. Oryza2000 used organ N_c_ curves in relation to development time based on a small dataset, which is empirical and has high uncertainty in nitrogen modeling. This study develops new organ N_c_ curves using a multi-source dataset, which could be useful for the application of wheat models. The uncertainty of the N_c_ dilution curve in the crop model and the theory of N distribution warrant further study.

## Conclusion

This study investigated the uncertainty and drivers of organ-specific critical nitrogen dilution curves under different conditions. By using hierarchical Bayesian theory, parameters A_1_ and A_2_ of the organ-specific N_c_ dilution curves for wheat were derived and evaluated under 14 different G × E × M N fertilizer experiments. The uncertainty of the N_c_ dilution curve was related to biomass and LAI level. Although the variation of parameter A_1_ was less than that of A_2_, the values of both parameters can change significantly in response to G × E × M. The drivers include maximum biomass, duration, and AGDD during the vegetative growth period and planting density. NNI calculated using organ-specific N_c_ is generally consistent with NNI estimated with overall shoot N_c_, indicating that a simple organ-specific N_c_ dilution curve may be used for wheat N diagnosis to assist N management. However, the significant differences in organ-specific N_c_ dilution curves across G × E × M conditions imply potential errors in N_c_ and crop N demand estimated using a general N_c_ dilution curve in crop models, highlighting a clear need for improvement in N_c_ calculations in such models. Our results provide new insights into how to improve modeling of crop nitrogen–biomass relations and N management practices under G × E × M.

## Data Availability

The datasets analyzed in this study are available from the corresponding authors upon reasonable request.
