# Supplementary material for: Interaction of Genotype, Environment, and Management on Organ-Specific Critical Nitrogen Dilution Curve in Wheat
Source: Plant Phenomics. 2023 Aug 2;5:0078. doi: 10.34133/plantphenomics.0078 (PMC10396079; doi:10.34133/plantphenomics.0078)
Supplement: Supplementary 1 — Table S1 [file plantphenomics.0078.f1.doc]

Table S1: General descriptions of the field experiments

| Exp. No | Exp. abbreviation | Year | Location | Cultivar | N rate (Kg ha^-1^) | N topdressing rate (%) | Sowing date | Plant density  (×10^4^ plants ha^-1^) | Row spacing  (m) |
| --- | --- | --- | --- | --- | --- | --- | --- | --- | --- |
| 1 | 2010-YZ-YM16 | 2009-2010 | Yizheng | Yangmai 16 | 0-75-150-225-300 | 50 | Nov 5, 2009 | 180 | 0.25 |
| 2 | 2010-YZ-NM13 | 2009-2010 | Yizheng | Ningmai 13 | 0-75-150-225-300 | 50 | Nov 5, 2009 | 180 | 0.25 |
| 3 | 2011-YZ-YM16 | 2010-2011 | Yizheng | Yangmai 16 | 0-75-150-225-300-375 | 50 | Nov 6, 2010 | 180 | 0.25 |
| 4 | 2011-YZ-NM13 | 2010-2011 | Yizheng | Ningmai 13 | 0-75-150-225-300-375 | 50 | Nov 6, 2010 | 180 | 0.25 |
| 5 | 2011-YZ-YM16* | 2010-2011 | Yizheng | Yangmai 16 | 0-225-300 | 70-60-50-40-30 | Oct 18, 2010 | 240 | 0.25 |
| 6 | 2012-XX-AK58 | 2011-2012 | Xinxiang | Aikang 58 | 0-75-150-225-300 | 65-50-35 | Nov 7, 2011 | 300 | 0.25 |
| 7 | 2013-RG-XM30 | 2012-2013 | Rugao | Xumai 30 | 0-75-150-225-300 | 50 | Oct 28, 2012 | 225 | 0.25 |
| 8 | 2013-RG-NM13 | 2012-2013 | Rugao | Ningmai 13 | 0-75-150-225-300 | 50 | Oct 28, 2012 | 225 | 0.25 |
| 9 | 2014-RG-XM30 | 2013-2014 | Rugao | Xumai 30 | 0-75-150-225-300 | 50 | Oct 26, 2013 | 225 | 0.25 |
| 10 | 2014-RG-NM13 | 2013-2014 | Rugao | Ningmai 13 | 0-75-150-225-300 | 50 | Oct 26, 2013 | 225 | 0.25 |
| 11 | 2014-XZ-XM30 | 2013-2014 | Xuzhou | Xumai 30 | 0-90-180-270-375 | 50 | Oct 26, 2013 | 240 | 0.20 |
| 12 | 2014-XZ-JM22 | 2013-2014 | Xuzhou | Jimai 22 | 0-90-180-270-375 | 50 | Oct 26, 2013 | 240 | 0.20 |
| 13 | 2016-SH-XM30 | 2015-2016 | Sihong | Xumai 30 | 0-90-180-270-360 | 50 | Oct 23, 2015 | 225 | 0.25 |
| 14 | 2016-SH-HM20 | 2015-2016 | Sihong | Huaimai 20 | 0-90-180-270-360 | 50 | Oct 23, 2015 | 225 | 0.25 |

Table S2: Geographical locations and initial soil conditions of the experimental sites

| Year | Location | Cultivar | Soil characteristics |
| --- | --- | --- | --- |
| 2009-2010 | Yizheng | Yangmai 16 | Organic matter: 18.94 g kg^-1^ |
|  | 119°10' E, 32°16' N | Ningmai 13 | Total N: 1.51 g kg^-1^ |
|  |  |  | Available N: 100.00 mg kg^-1^ |
|  |  |  | Available P: 34.34 mg kg^-1^ |
|  |  |  | Available K: 90.75 mg kg^-1^ |
| 2010-2011 | Yizheng | Yangmai 16 | Organic matter: 13.54 g kg^-1^ |
|  | 119°10' E, 32°16' N | Ningmai 13 | Total N: 1.23 g kg^-1^ |
|  |  |  | Available N: 66.00 mg kg^-1^ |
|  |  |  | Available P: 43.43 mg kg^-1^ |
|  |  |  | Available K: 82.04 mg kg^-1^ |
| 2011-2012 | Xinxiang | Aikang 58 | Organic matter: 15.70 g kg^-1^ |
|  | 113°48 ′E, 35°11′N |  | Total N: 1.45 g kg^-1^ |
|  |  |  | Available N: - |
|  |  |  | Available P: 67.54 mg kg^-1^ |
|  |  |  | Available K: 87.43 mg kg^-1^ |
| 2012-2013 | Rugao | Xumai 30 | Organic matter: 30.5 g kg^-1^ |
|  | 120°76′E, 32°27 ′N | Ningmai 13 | Total N: 2.49 g kg^-1^ |
|  |  |  | Available N: 170.48 mg kg^-1^ |
|  |  |  | Available P: 52.63 mg kg^-1^ |
|  |  |  | Available K: 93.48 mg kg^-1^ |
| 2013-2014 | Rugao | Xumai 30 | Organic matter: 24.6 g kg^-1^ |
|  | 120°76′E, 32°27 ′N | Ningmai 13 | Total N: 1.87 g kg^-1^ |
|  |  |  | Available N: 150.41 mg kg^-1^ |
|  |  |  | Available P: 57.84 mg kg^-1^ |
|  |  |  | Available K: 96.32 mg kg^-1^ |
| 2013-2014 | Xuzhou | Xumai 30 | Organic matter: 35.5 g kg^-1^ |
|  | 117°13′E, 34°47 ′N | Jimai 22 | Total N: 1.55 g kg^-1^ |
|  |  |  | Available N: 122.53 mg kg^-1^ |
|  |  |  | Available P: 45.83 mg kg^-1^ |
|  |  |  | Available K: 80.72 mg kg^-1^ |
| 2015-2016 | Sihong | Xumai 30 | Organic matter: 26.5 g kg^-1^ |
|  | 118°26′E, 33°36′N | Huaimai 20 | Total N: 2.07 g kg^-1^ |
|  |  |  | Available N:160.26 mg kg^-1^ |
|  |  |  | Available P: 52.45 mg kg^-1^ |
|  |  |  | Available K: 93.66 mg kg^-1^ |

Table S3: Sampling dates in the experiments

| Exp. No | Exp. abbreviation | Shoot biomass sampling date  (days after sowing) | Leaf and stem biomass sampling date  (days after sowing) | Leaf area index sampling date  (days after sowing) |
| --- | --- | --- | --- | --- |
| 1 | 2010-YZ-YM16 | 140, 158, 164, 174, 185, 200, 207 | 140, 158, 164, 174, 185 | 140, 158, 164, 174, 185, 200, 207 |
| 2 | 2010-YZ-NM13 | 140, 158, 164, 174, 185, 200, 207 | 140, 158, 164, 174, 185 | 140, 158, 164, 174, 185, 200, 207 |
| 3 | 2011-YZ-YM16 | 117, 137, 143, 152, 159, 170, 176 | 117, 137, 143, 152, 159, 170, 176 | 117, 137, 143, 152, 159, 170, 176, 184, 193 |
| 4 | 2011-YZ-NM13 | 117, 137, 143, 152, 159, 170, 176 | 117, 137, 143, 152, 159, 170, 176 | 117, 137, 143, 152, 159, 170, 176, 184, 193 |
| 5 | 2011-YZ-YM16* | 138, 144, 153, 160, 171 | 138, 144, 153, 160, 171 | 138, 144, 153, 160, 171 |
| 6 | 2012-XX-AK58 | 124, 139, 147, 154, 162, 170, 175 | 124, 139, 147, 154, 162, 170, 175 |  |
| 7 | 2013-RG-XM30 | 119, 132, 142, 153, 161, 166 | 119, 132, 142, 153, 161, 166 | - |
| 8 | 2013-RG-NM13 | 119, 132, 142, 153, 161, 166 | 119, 132, 142, 153, 161, 166 | - |
| 9 | 2014-RG-XM30 | 111, 121, 134, 140, 152, 160, 166, 171, 178 | 111, 121, 134, 140, 152, 160, 166, 171, 178 | 121, 134, 140, 152, 160, 166, 171, 178 |
| 10 | 2014-RG-NM13 | 111, 121, 134, 140, 152, 160, 166, 171, 178 | 121, 134, 140, 152, 160, 166, 171, 178 | 121, 134, 140, 152, 160, 166, 171, 178 |
| 11 | 2014-XZ-XM30 | 129, 145, 158, 166, 176 | 129, 145, 158, 166, 176 | - |
| 12 | 2014-XZ-JM22 | 129, 145, 158, 166, 176 | 129, 145, 158, 166, 176 | - |
| 13 | 2016-SH-XM20 | 153, 166, 171, 175, 183, 187 | 133, 146, 153, 166, 171, 175, 183, 187, 196 | 133, 146, 153, 166, 171, 175, 183, 187, 196 |
| 14 | 2016-SH-HM20 | 153, 166, 171, 175, 183, 187 | 133, 146, 153, 166, 171, 175, 183, 187, 196 | 133, 146, 153, 166, 171, 175, 183, 187, 196 |

Table S4. The posterior distribution (quantiles) of curve parameters A_1_ for wheat leaf biomass across different G × E × M conditions.

| Exp. No | Exp. abbreviation | A_1_ | | | | | A_2_ | | | | |
| --- | --- | --- | --- | --- | --- | --- | --- | --- | --- | --- | --- |
|  |  | 2.50% | 25% | 50% | 75% | 97.50% | 2.50% | 25% | 50% | 75% | 97.50% |
| 1 | 2010-YZ-YM16 | 2.75 | 2.85 | 2.92 | 2.99 | 3.19 | 0.07 | 0.13 | 0.16 | 0.20 | 0.29 |
| 2 | 2010-YZ-NM13 | 2.78 | 2.86 | 2.92 | 3.00 | 3.18 | 0.02 | 0.07 | 0.10 | 0.13 | 0.21 |
| 3 | 2011-YZ-YM16 | 2.98 | 3.16 | 3.24 | 3.33 | 3.54 | 0.13 | 0.19 | 0.23 | 0.26 | 0.33 |
| 4 | 2011-YZ-NM13 | 2.71 | 2.81 | 2.87 | 2.93 | 3.06 | 0.03 | 0.09 | 0.12 | 0.15 | 0.21 |
| 5 | 2011-YZ-YM16* | 3.07 | 3.18 | 3.23 | 3.30 | 3.45 | 0.03 | 0.09 | 0.13 | 0.17 | 0.27 |
| 6 | 2012-XX-AK58 | 3.18 | 3.25 | 3.28 | 3.32 | 3.40 | 0.06 | 0.10 | 0.12 | 0.14 | 0.17 |
| 7 | 2013-RG-XM30 | 3.33 | 3.68 | 4.07 | 4.76 | 9.01 | 0.02 | 0.12 | 0.22 | 0.39 | 1.02 |
| 8 | 2013-RG-NM13 | 3.55 | 4.06 | 4.46 | 5.10 | 7.69 | 0.09 | 0.23 | 0.32 | 0.46 | 0.85 |
| 9 | 2014-RG-XM30 | - | - | - | - | - | - | - | - | - | - |
| 10 | 2014-RG-NM13 | - | - | - | - | - | - | - | - | - | - |
| 11 | 2014-XZ-XM30 | 2.87 | 3.06 | 3.18 | 3.32 | 3.70 | 0.00 | 0.04 | 0.09 | 0.15 | 0.30 |
| 12 | 2014-XZ-JM22 | 3.06 | 3.24 | 3.34 | 3.47 | 3.80 | 0.00 | 0.03 | 0.05 | 0.10 | 0.22 |
| 13 | 2016-SH-XM30 | 3.03 | 3.18 | 3.26 | 3.36 | 3.61 | 0.00 | 0.03 | 0.06 | 0.11 | 0.24 |
| 14 | 2016-SH-HM20 | 3.27 | 3.41 | 3.51 | 3.61 | 3.79 | 0.00 | 0.01 | 0.03 | 0.05 | 0.13 |

Table S5. The posterior distribution (quantiles) of curve parameters A_2_ for wheat stem biomass across different G × E × M conditions.

| Exp. No | Exp. abbreviation | A_1_ | | | | | A_2_ | | | | |
| --- | --- | --- | --- | --- | --- | --- | --- | --- | --- | --- | --- |
|  |  | 2.50% | 25% | 50% | 75% | 97.50% | 2.50% | 25% | 50% | 75% | 97.50% |
| 1 | 2010-YZ-YM16 | 2.19 | 2.50 | 2.67 | 2.83 | 3.21 | 0.15 | 0.26 | 0.31 | 0.37 | 0.48 |
| 2 | 2010-YZ-NM13 | 2.45 | 2.76 | 2.91 | 3.07 | 3.40 | 0.20 | 0.33 | 0.39 | 0.45 | 0.57 |
| 3 | 2011-YZ-YM16 | 2.83 | 3.42 | 3.80 | 4.27 | 5.54 | 0.36 | 0.51 | 0.59 | 0.68 | 0.86 |
| 4 | 2011-YZ-NM13 | 2.08 | 2.44 | 2.64 | 2.86 | 3.44 | 0.22 | 0.34 | 0.40 | 0.46 | 0.60 |
| 5 | 2011-YZ-YM16* | 2.12 | 2.27 | 2.35 | 2.44 | 2.63 | 0.42 | 0.49 | 0.52 | 0.55 | 0.62 |
| 6 | 2012-XX-AK58 | 2.00 | 2.08 | 2.12 | 2.17 | 2.26 | 0.40 | 0.43 | 0.45 | 0.46 | 0.50 |
| 7 | 2013-RG-XM30 | 2.22 | 2.57 | 2.78 | 3.03 | 3.56 | 0.45 | 0.53 | 0.57 | 0.62 | 0.71 |
| 8 | 2013-RG-NM13 | 2.46 | 2.70 | 2.84 | 2.99 | 3.38 | 0.36 | 0.43 | 0.47 | 0.52 | 0.63 |
| 9 | 2014-RG-XM30 | 1.62 | 1.80 | 1.91 | 2.02 | 2.27 | 0.25 | 0.30 | 0.33 | 0.36 | 0.43 |
| 10 | 2014-RG-NM13 | 1.77 | 2.06 | 2.20 | 2.34 | 2.68 | 0.31 | 0.43 | 0.48 | 0.52 | 0.60 |
| 11 | 2014-XZ-XM30 | 1.59 | 1.88 | 2.05 | 2.21 | 2.50 | 0.15 | 0.23 | 0.27 | 0.31 | 0.36 |
| 12 | 2014-XZ-JM22 | 1.55 | 1.77 | 1.88 | 1.99 | 2.24 | 0.19 | 0.26 | 0.30 | 0.34 | 0.46 |
| 13 | 2016-SH-XM30 | 1.85 | 2.10 | 2.26 | 2.45 | 2.93 | 0.13 | 0.21 | 0.26 | 0.32 | 0.43 |
| 14 | 2016-SH-HM20 | 1.71 | 1.91 | 2.03 | 2.16 | 2.49 | 0.15 | 0.21 | 0.24 | 0.29 | 0.41 |

Table S6. The posterior distribution (quantiles) of curve parameters A_2_ for wheat leaf area index (LAI) across different G × E × M conditions.

| Exp. No | Exp. abbreviation | A_1_ | | | | | A_2_ | | | | |
| --- | --- | --- | --- | --- | --- | --- | --- | --- | --- | --- | --- |
|  |  | 2.50% | 25% | 50% | 75% | 97.50% | 2.50% | 25% | 50% | 75% | 97.50% |
| 1 | 2010-YZ-YM16 | 2.38 | 2.46 | 2.53 | 2.60 | 2.76 | 0.16 | 0.19 | 0.20 | 0.22 | 0.27 |
| 2 | 2010-YZ-NM13 | 0.16 | 0.19 | 0.20 | 0.22 | 0.27 | 0.16 | 0.19 | 0.20 | 0.22 | 0.27 |
| 3 | 2011-YZ-YM16 | 3.03 | 3.27 | 3.41 | 3.55 | 3.83 | 0.26 | 0.31 | 0.33 | 0.36 | 0.41 |
| 4 | 2011-YZ-NM13 | 3.07 | 3.32 | 3.47 | 3.62 | 3.95 | 0.27 | 0.33 | 0.36 | 0.39 | 0.45 |
| 5 | 2011-YZ-YM16* | - | - | - | - | - | - | - | - | - | - |
| 6 | 2012-XX-AK58 | - | - | - | - | - | - | - | - | - | - |
| 7 | 2013-RG-XM30 | - | - | - | - | - | - | - | - | - | - |
| 8 | 2013-RG-NM13 | - | - | - | - | - | - | - | - | - | - |
| 9 | 2014-RG-XM30 | 3.07 | 4.52 | 5.89 | 7.99 | 11.44 | 0.31 | 0.55 | 0.70 | 0.88 | 1.09 |
| 10 | 2014-RG-NM13 | 2.60 | 3.19 | 3.69 | 4.45 | 8.60 | 0.13 | 0.25 | 0.33 | 0.45 | 0.84 |
| 11 | 2014-XZ-XM30 | - | - | - | - | - | - | - | - | - | - |
| 12 | 2014-XZ-JM22 | - | - | - | - | - | - | - | - | - | - |
| 13 | 2016-SH-XM30 | 2.73 | 3.09 | 3.30 | 3.55 | 4.18 | 0.19 | 0.29 | 0.33 | 0.39 | 0.53 |
| 14 | 2016-SH-HM20 | 2.73 | 3.16 | 3.48 | 3.93 | 5.84 | 0.29 | 0.42 | 0.51 | 0.63 | 1.00 |

Table S7. The posterior distribution (quantiles) of curve parameters A_2_ for wheat shoot biomass across different G × E × M conditions.

| Exp. No | Exp. abbreviation | A_1_ | | | | | A_2_ | | | | |
| --- | --- | --- | --- | --- | --- | --- | --- | --- | --- | --- | --- |
|  |  | 2.50% | 25% | 50% | 75% | 97.50% | 2.50% | 25% | 50% | 75% | 97.50% |
| 1 | 2010-YZ-YM16 | 3.28 | 3.80 | 4.11 | 4.42 | 5.06 | 0.26 | 0.34 | 0.38 | 0.42 | 0.50 |
| 2 | 2010-YZ-NM13 | 3.57 | 4.02 | 4.28 | 4.58 | 5.23 | 0.33 | 0.41 | 0.45 | 0.50 | 0.58 |
| 3 | 2011-YZ-YM16 | 3.93 | 4.35 | 4.57 | 4.79 | 5.33 | 0.35 | 0.41 | 0.44 | 0.47 | 0.53 |
| 4 | 2011-YZ-NM13 | 3.49 | 3.87 | 4.06 | 4.24 | 4.59 | 0.34 | 0.40 | 0.43 | 0.46 | 0.50 |
| 5 | 2011-YZ-YM16* | 3.35 | 3.62 | 3.78 | 3.94 | 4.32 | 0.33 | 0.40 | 0.43 | 0.46 | 0.52 |
| 6 | 2012-XX-AK58 | 3.44 | 3.61 | 3.71 | 3.81 | 4.04 | 0.34 | 0.37 | 0.39 | 0.41 | 0.45 |
| 7 | 2013-RG-XM30 | 3.75 | 4.57 | 5.23 | 6.10 | 8.64 | 0.33 | 0.44 | 0.51 | 0.58 | 0.75 |
| 8 | 2013-RG-NM13 | 3.54 | 4.05 | 4.48 | 5.17 | 7.00 | 0.20 | 0.29 | 0.35 | 0.41 | 0.55 |
| 9 | 2014-RG-XM30 | 3.06 | 3.53 | 3.80 | 4.11 | 4.97 | 0.28 | 0.35 | 0.39 | 0.43 | 0.53 |
| 10 | 2014-RG-NM13 | 3.24 | 3.70 | 4.01 | 4.38 | 5.51 | 0.29 | 0.37 | 0.42 | 0.47 | 0.63 |
| 11 | 2014-XZ-XM30 | 2.80 | 3.29 | 3.54 | 3.81 | 4.38 | 0.24 | 0.31 | 0.35 | 0.38 | 0.44 |
| 12 | 2014-XZ-JM22 | 2.91 | 3.35 | 3.58 | 3.82 | 4.33 | 0.30 | 0.36 | 0.39 | 0.42 | 0.48 |
| 13 | 2016-SH-XM30 | 2.06 | 2.36 | 2.70 | 3.30 | 6.86 | 0.01 | 0.08 | 0.15 | 0.26 | 0.65 |
| 14 | 2016-SH-HM20 | 2.24 | 2.74 | 3.10 | 3.53 | 4.80 | 0.08 | 0.21 | 0.28 | 0.36 | 0.53 |


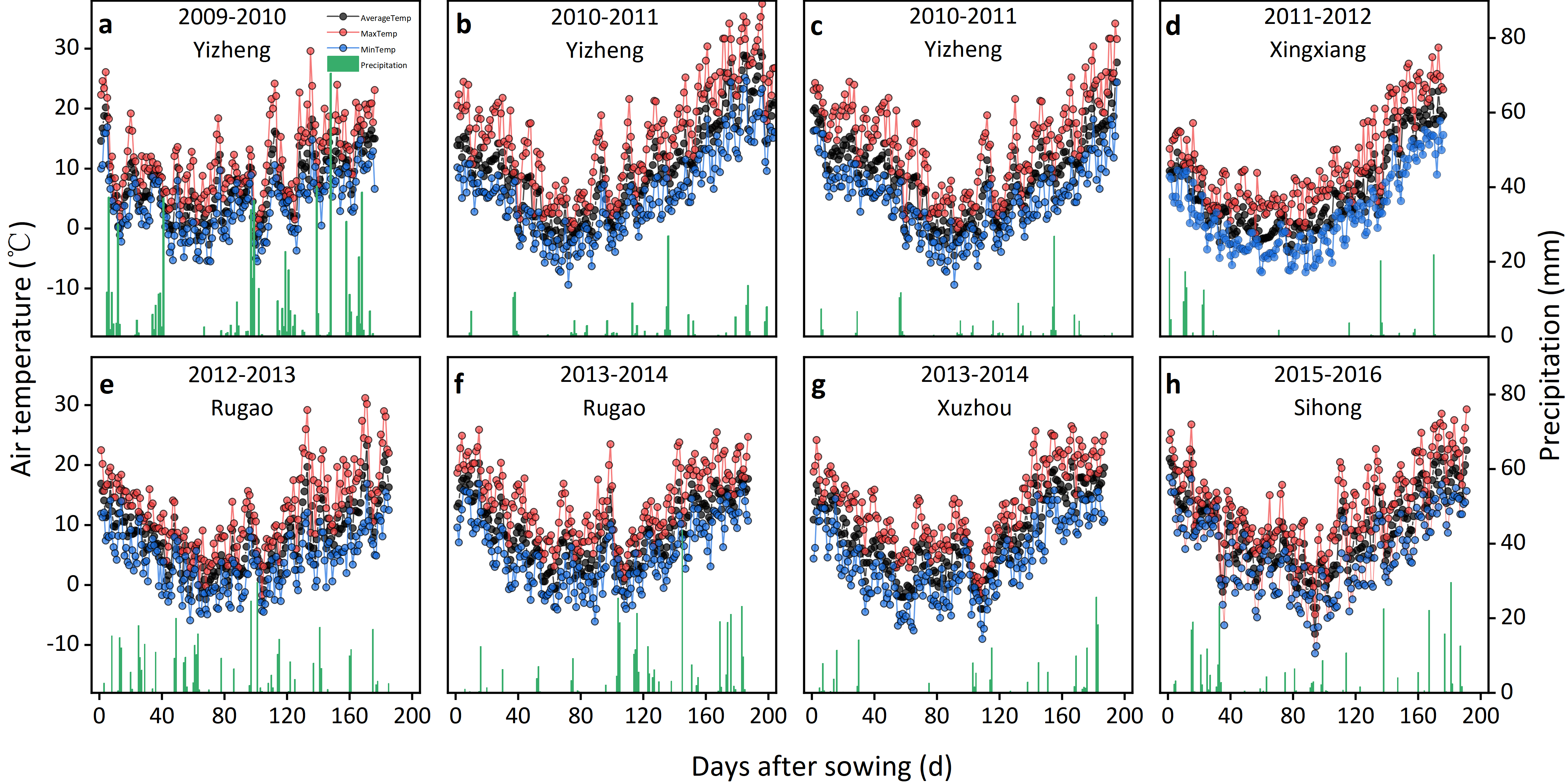


**Figure S1**. Air temperature and precipitation of each experiment.


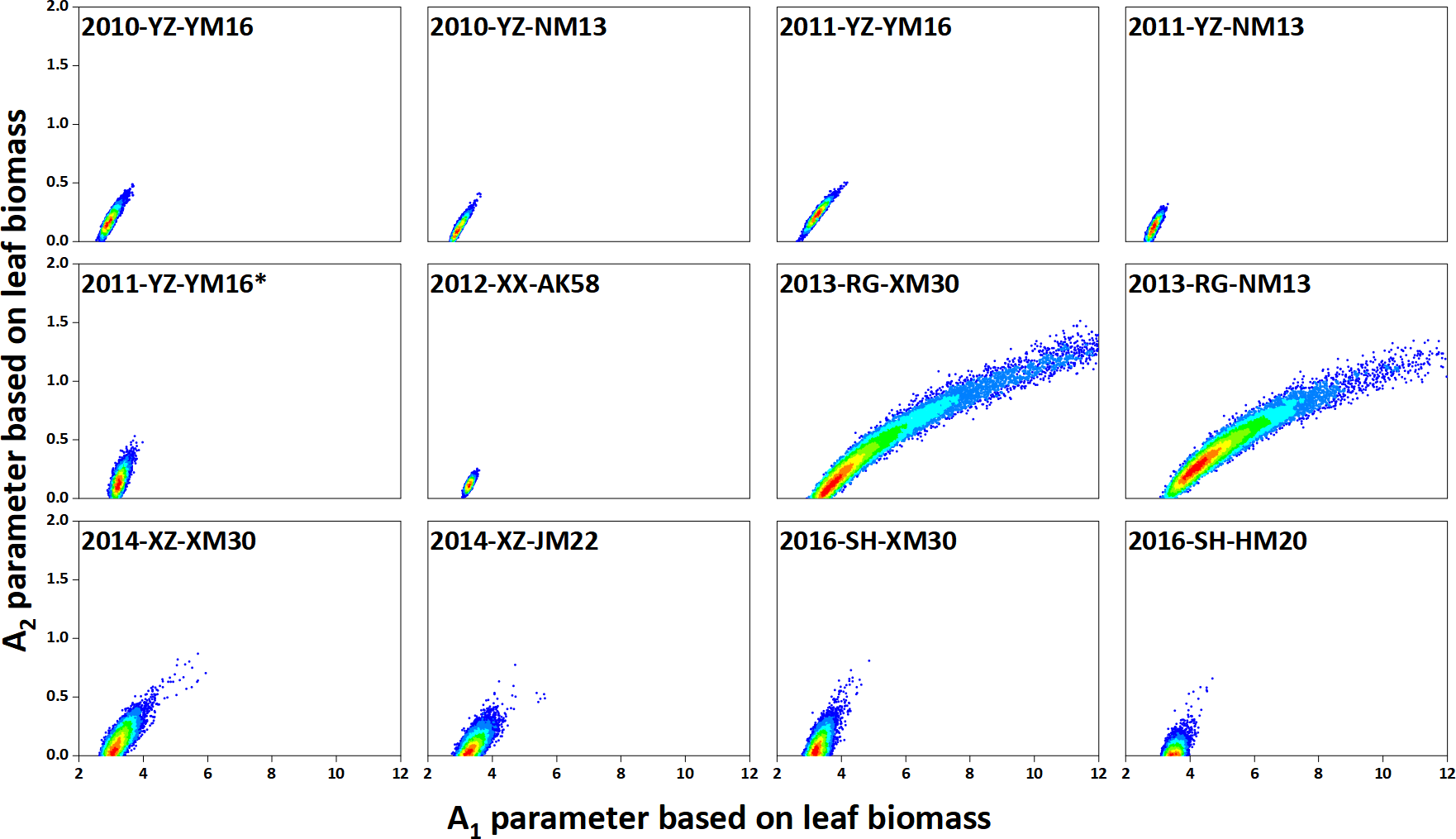


**Figure S2.** Cloud of fitted curve parameters A_1_ and A_2_ for leaf biomass basis.


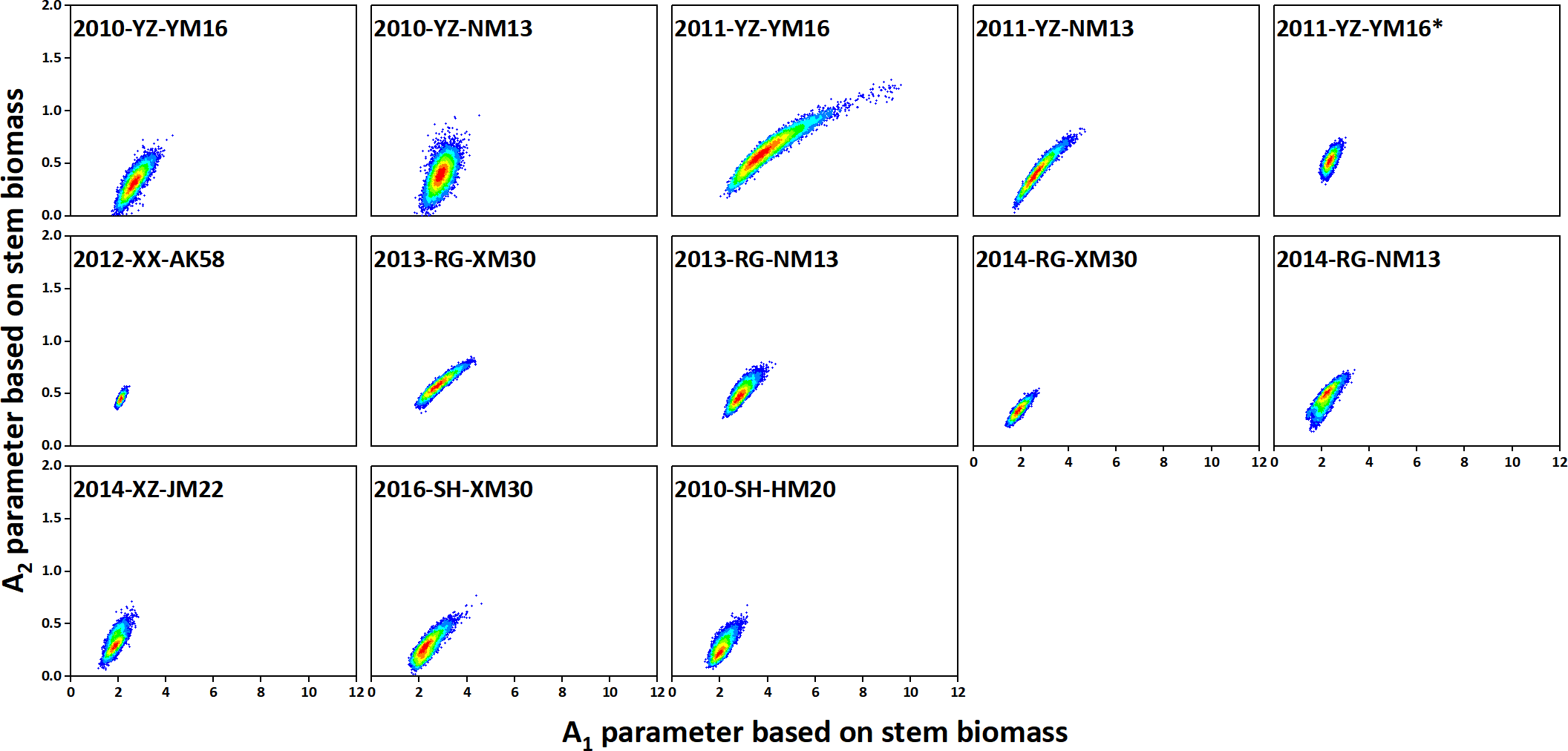


**Figure S3.** Cloud of critical nitrogen dilution curve parameters A_1_ and A_2_ for stem biomass basis.


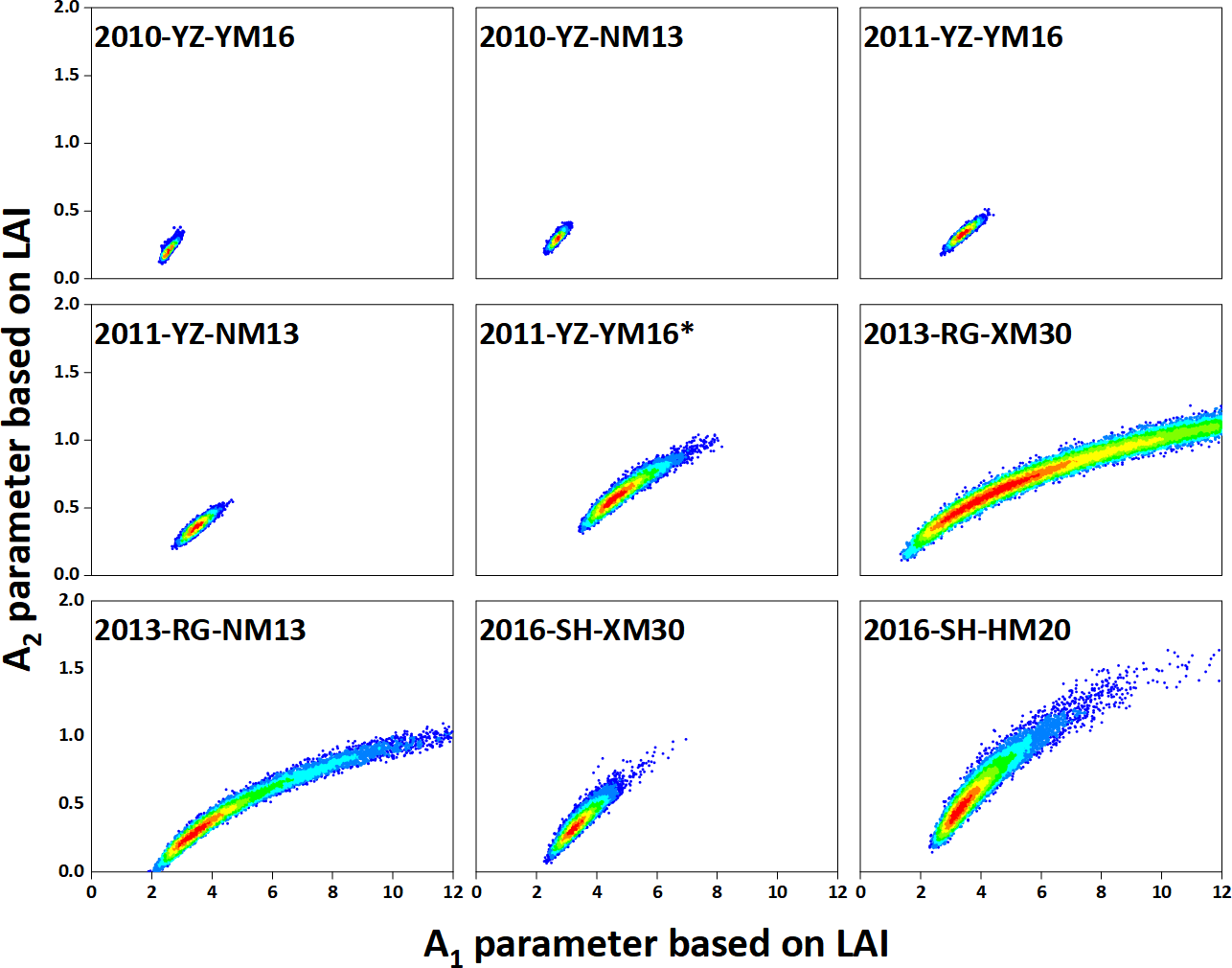


**Figure S4.** Cloud of critical nitrogen dilution curve parameters A_1_ and A_2_ for leaf area index (LAI) basis.


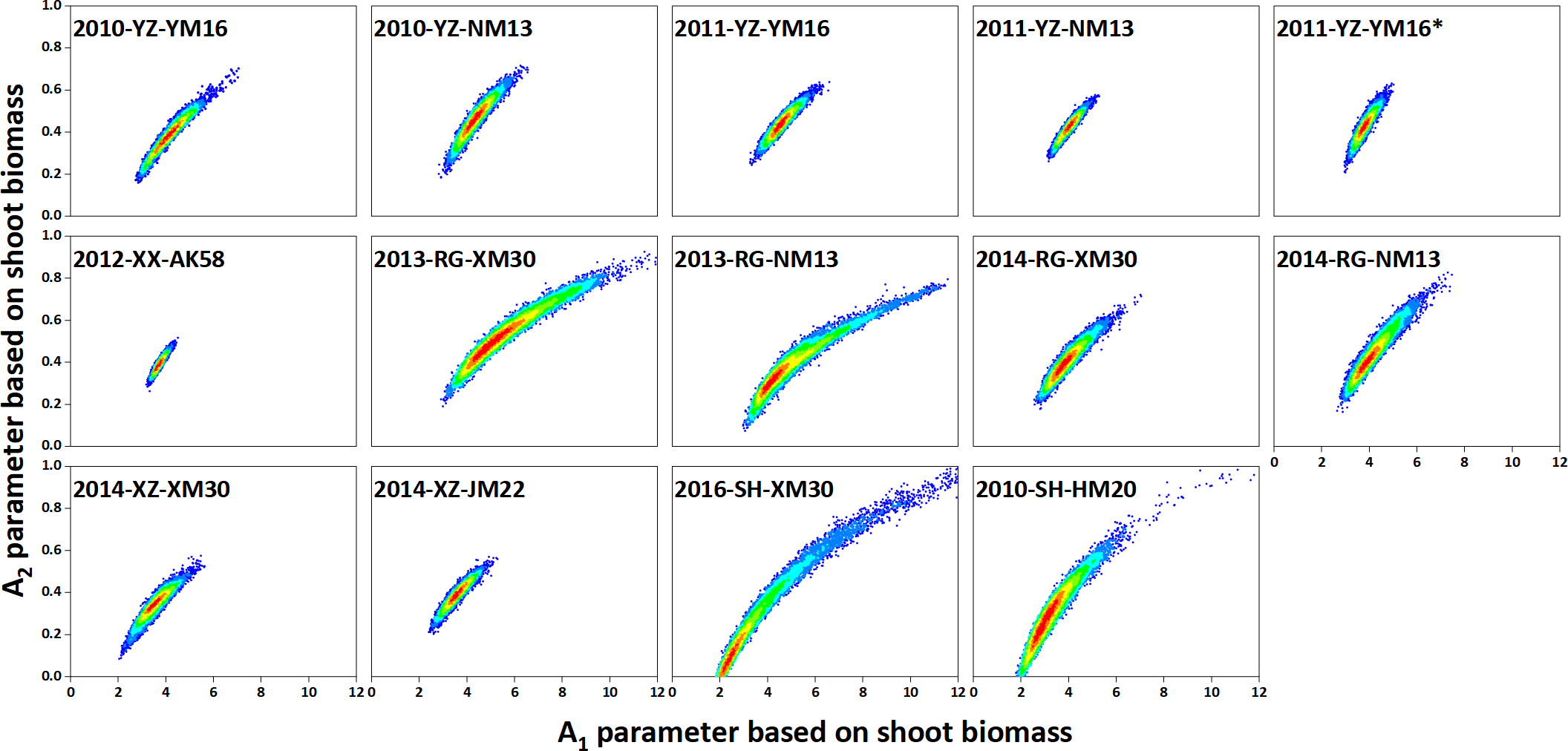


**Figure S5.** Cloud of critical nitrogen dilution curve parameters A_1_ and A_2_ for shoot biomass basis.


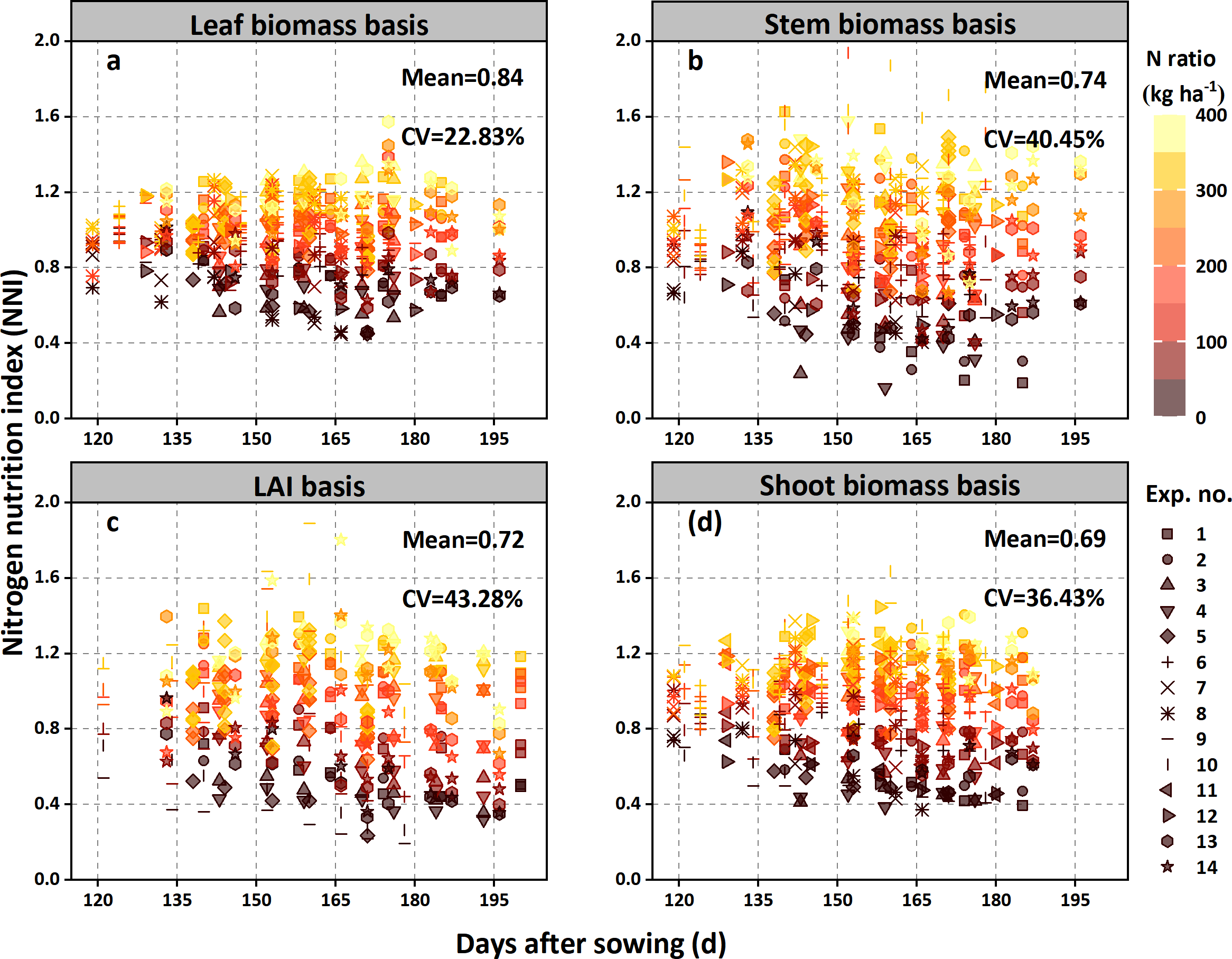


**Figure S6.** N nutrition index derived from different wheat organs under different N treatments (panel a: leaf biomass; panel b: stem biomass; panel c: leaf area index; panel d: shoot biomass).
